# Applications of magnetic resonance spectroscopy in diagnosis of neurodegenerative diseases: A systematic review

**DOI:** 10.1016/j.heliyon.2024.e30521

**Published:** 2024-04-30

**Authors:** Fatemeh Abbaspour, Niusha Mohammadi, Hassan Amiri, Susan Cheraghi, Reza Ahadi, Zeinab Hormozi-Moghaddam

**Affiliations:** a Department of Radiation Sciences, Allied Medicine Faculty, Iran University of Medical Sciences, Tehran, Iran; b Emergency Medicine Management Research Center, Iran University of Medical Sciences, Tehran, Iran; c Radiation Biology Research Center, Iran University of Medical Sciences, Tehran, Iran; d Department of Anatomical Science Iran University of Medical Science Tehran, Iran

**Keywords:** Magnetic resonance spectroscopy, Neurodegenerative, Diagnosis

## Abstract

**Background:**

Magnetic resonance spectroscopy (MRS) is an imaging technique used to measure metabolic changes in the tissue. Due to the lack of evidence, MRS is not a priority in diagnosing neurodegenerative diseases because it is a relatively specialized technique that requires specialized equipment and expertise to perform and interpret. This systematic review aimed to present a comprehensive collection of MRS results in the most common neurodegenerative diseases.

**Methods:**

A systematic search of four electronic databases (PubMed, Scopus, Web of Science, and ScienceDirect) was conducted for studies published from 2017 to 2022. Articles that provided specific biomarker levels were selected, and studies that assessed the diseases via treatment, featured MRS applying nuclei other than 1H, or compared different animal models were excluded.

**Results:**

A total of 25 articles, plus 3 articles for extra information in the introduction, were included in this review. Six of the most common neurodegenerative diseases, i.e., Alzheimer's and Parkinson's disease, Huntington chorea, ataxia, multiple sclerosis (MS), multiple system atrophy (MSA), and progressive supranuclear palsy (PSP) were examined via MRS. The changes and ratios of N-acetylaspartate (NAA) could be seen in all of these disorders, which could lead to early diagnosis. However, there are other biomarkers, such as Cr and Chon, which can give convincing results.

**Discussion:**

This observational study is the first synthesis of the latest evidence proving metabolic changes during neurodegenerative diseases using MRS as a diagnosis method. The findings indicate decreased N-acetylaspartate (NAA) and NAA/Cr ratios in Alzheimer's disease (AD), Parkinson's disease (PD), ataxias, and MS, reflecting neuronal loss or dysfunction. Increased choline and myo-inositol were noted in some studies, suggesting cell membrane turnover and neuroinflammation. Findings were less consistent for other metabolites like glutamate and gamma-aminobutyric acid. However, there were limitations due to the lack of studies on the same volumes of interest (VOIs) and the small number of participants.

## 1 Introduction

Neurodegenerative diseases are characterized by a slow degeneration of the neuronal structure, leading to cognitive, psychiatric, and motor symptoms [1]. One method to assess the concentration of specific molecules in living tissue is in-vivo proton magnetic (or nuclear) resonance spectroscopy (1H-MRS). MRS works with electromagnetic waves to measure biomedical/metabolic information and provides access to hydrogen nuclear spins in magnetic fields [2]. This non-invasive method allows non-invasive measurements of neurochemicals in either single voxel or multiple voxels. Single-voxel MRS (SVS) techniques produce MR spectra from a single VOI, whereas multi-voxel MRS techniques produce spectra from many neighboring volumes, often covering a greater area than SVS [3]. Single-voxel MRS uses a single region of interest (ROI) to provide detailed information about metabolite concentrations within that region but does not reveal spatial variations across the entire tissue or organ. On the other hand, multi-voxel MRS selects multiple regions of interest and acquires spectra from each voxel within a two- or three-dimensional grid. This allows for simultaneous investigation of multiple regions and reveals metabolic heterogeneity and variations in metabolite concentrations (which may be important for understanding disease-related alterations). The stimulated echo acquisition mode (STEAM), point-resolved spectroscopy (PRESS), image-selected in vivo spectroscopy (ISIS), and outer volume suppression (OVS) are all common SVS techniques. Multi-voxel MRS techniques are image-based localization methods, also known as magnetic resonance spectroscopic imaging (MRSI), chemical-shift imaging (CSI), or simply spectroscopic imaging (SI) [3].

### 1.1 Alzheimer's disease (AD)

Alzheimer's disease (AD) is a neurodegenerative disorder that gradually advances, leading to the death of brain nerve cells and, eventually, severe dementia [2]. The disease has various stages, of which five are currently known, and mild cognitive impairment (MCI) has been considered an intermediate stage between the normal state and AD [4].

### 1.2 Ataxia

Ataxia is a commonly encountered clinical presentation in neurology clinics, resulting from many hereditary and non-hereditary causes [5]. Autosomal dominant inherited spinocerebellar ataxias (SCA) and autosomal recessive Friedreich ataxia (FA) belong to the most frequent progressive neurodegenerative ataxias [6]. SCA consists of several (approximately 40 known so far) types of hereditary, progressive autosomal dominant heterogeneous neurodegenerative diseases. SCAs primarily affect the cerebellum and spinal cord but may also cause degeneration in other parts of the central nervous system [7]. Gluten ataxia (GA), a rare immune-mediated cerebellar ataxia (IMCA), is a neurological presentation of gluten sensitivity enteropathy and can be demonstrated (although non-specifically) through the concentration of antigliadin antibodies in the blood serum [8].

### 1.3 Huntington's disease

Huntington's disease (HD) is a polyglutamine disorder caused by an expansion in the glutamine encoding cytosine-adenine-guanine (CAG) repeats, and striatal atrophy is a prominent characteristic of the disease. Other brain regions, including the thalamus, cerebral cortex, cerebellum, and visual cortex, are also involved in the pathological process as the disease progresses [9].

### 1.4 Parkinson's disease (PD)

Parkinson's disease (PD) is a predominantly age-related neurodegenerative disorder of the brain. It causes the deterioration of dopamine-producing neurons in the basal ganglia (the nuclei, including substantia nigra, and globus pallidus), gradually progressing to the neocortex [10].

### 1.5 Multiple sclerosis (MS)

Multiple sclerosis (MS) is an autoimmune inflammatory disorder of the central nervous system. MS phenotypes are described as clinically isolated syndrome (CIS), relapsing-remitting multiple sclerosis (RRMS), and progressive multiple sclerosis (PMS). RRMS is characterized as active or non-active. PMS can be primary progressive (PP) or secondary progressive (SP) [11].

### 1.6 Multiple system atrophy (MSA)

Multiple system atrophy (MSA) is an uncommon neurodegenerative disease, which usually begins at 50–60 years of age but has been observed in ages as young as 30. It has two general subtypes of Parkinsonian (MSAp) and cerebellar (MSAc), with the former presenting similar to Parkinson disease and the latter associated with ataxia-like symptoms [12].

### 1.7 Progressive supranuclear palsy (PSP)

Progressive supranuclear palsy (PSP) is clinically defined as a progressive neurodegenerative disease associated with axial rigidity, bradykinesia, postural instability, vertical supranuclear gaze palsy, speech and swallowing dysfunctions, as well as front executive and behavioral manifestations [13].

### 1.8 Small-molecule diagnostic biomarkers

**N-acetylaspartate (NAA)**: NAA is a small-molecule (molecular weight: 175.139) metabolite synthesized in neuron mitochondria from aspartate and acetyl-coenzyme A. It has various functions, including osmoregulation and acetate storage for the synthesis of lipids and myelin. The NNA molecule has extremely high concentrations in the brain and creates the largest signal in an MRI scan, and abnormal NNA concentrations can indicate various neuropathologies [2].

**N-Acetylaspartylglutamate (NAAG)**: N N-acetylaspartylglutamate (NAAG) is a neuropeptide composed of glutamic acid and NAA. NAAG is a neuromodulator of glutamatergic transmissions, among others, and is found in extremely high amounts (millimolar scale) in the central nervous system [2].

**Creatine (Cr)**: Creatine (Cr) and its phosphorylated form creatine phosphate (CP) (or phosphocreatine (PCr)), together with creatine kinase enzyme, form a system that acts as an energy buffer by “shuttling” high-energy phosphate from adenosine triphosphate (ATP) to the cytoplasm of cells [2].

**Choline (Cho)**: Choline (or bilineurine) is a water-soluble nutrient found mainly as compounds in the form of alpha-GPC (α-glycerylphosphorylcholine) and phosphatidylcholine (PCho) in the cell membrane. Choline travels as free cations in the blood through the blood-brain barrier. It is a known precursor of acetylcholine (a neurotransmitter) synthesis and metabolism of essential phospholipids (such as phosphatidylcholine and sphingomyelin) [2].

**Inositol (Ins)**: Inositol is a carbocyclic polyalcohol with nine stereoisomers. The highest prevalence in biological tissue belongs to isomers myo-inositol (previously considered as vitamin B8), followed by scyllo-inositol. Myo-inositol is present freely in the blood and as phosphatidylinositol in cell membranes, particularly central nervous system tissues, and is a precursor of inositol lipid synthesis, thus playing an important role in cellular signal modulation and also as an osmolyte [2].

**Lactate (Lac)**: Lactate onion (lactic acid) is produced in the body during glucose metabolism, specifically as a byproduct of anaerobic glycolysis through the oxidization of glucose to pyruvate and its reduction to lactate. Various cells (particularly in brain tissue) metabolize lactate for energy. During exercise, there is a temporary increase in lactate in healthy individuals. However, a persistent excess of lactate is a pathological factor [2].

**Glutamate and Glutamine (Glu and Gln)**: Glutamate is the primary excitatory neurotransmitter of the central nervous system. Excess glutamate is recycled by astrocytes from the synapse after being transformed into glutamine. Glutamine is then sent back to the pre-synaptic neuron for glutamate synthesis. Total glutamate and glutamine levels in a brain region would be constant as long as the integrity of the neuron-astrocyte complex and an associated blood-CNS barrier is preserved [14].

**Gamma-aminobutyric acid**: γ-aminobutyric acid (GABA) is a non-proteinogenic inhibitory neurotransmitter and neuromodulator in the central nervous tissue. It is mainly synthesized from glutamate by glutamate decarboxylase (glutamic acid decarboxylase enzyme) and cofactor pyridoxal phosphate (active vitamin B6) [15].

This review aimed to collect up-to-date data on the role of MRS in diagnosing neurodegenerative diseases. Aging and higher life expectancy have increased the prevalence of these diseases, and they are considered significant public health problems in the coming years [1]. The early detection of degenerative diseases could significantly improve the treatment process. H-MRS is a promising tool for diagnosing these neurodegenerative diseases, and the science behind it is developing every day.

## 2 Methods

### 2.1 Search strategy and eligibility criteria

**Fig. 1:**
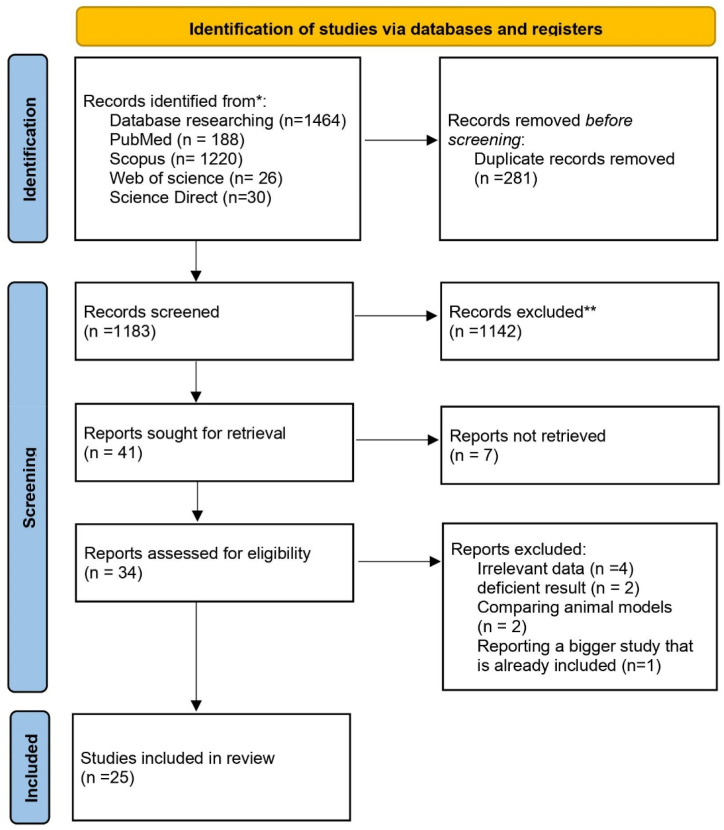
Study procedure.

**Fig. 2:**
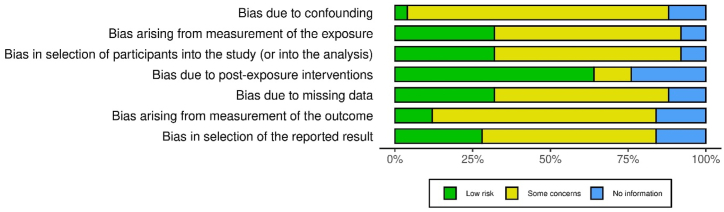
Risk of bias in studies.

We followed the Preferred Reporting Items for Systematic Review and Meta-Analysis (PRISMA) guidelines and performed a systematic review of MRS studies on neurodegenerative diseases published from 2017 to 2022 following a published protocol of a prospectively registered review (PROSPERO). The articles were searched on PubMed, Scopus, ScienceDirect, and Web of Science databases by two independent researchers in August 2022. The articles were restricted by time (2017–2022) and language (English). The search strategy used a combination of terms and free-text words in titles and abstracts, and terms related to MRS and neurodegenerative diseases were included (Additional File 1). The included articles involved imaging methods (e.g., MRI) in the diagnosis and evaluation of the diseases selected in the present study (i.e., AD, ataxia, HD, PSP, PD, and MS). Fig. 1 illustrates the study selection process for the systematic review. A total of 1464 studies were identified through database searches, and after screening for eligibility, 25 studies were included in the final analysis (see Fig. 2).

### 2.2 Study selection and data extraction

The reference manager software Mendeley was used to organize the selected studies and eliminate duplicate articles. The selection process consisted of two independent reviewers reading the titles and abstracts of articles to search for papers meeting the selection criteria.

### 2.3 Inclusion criteria

The reference manager software Mendeley was used to organize the selected studies and eliminate duplicate articles. The selection process consisted of two independent reviewers reading the titles and abstracts of articles to search for papers meeting the selection criteria.

### 2.4 Exclusion criteria

The following studies were excluded.

• Studies published before 2017.
• Studies that were not published in the English language.
• Studies that assessed the effect of medical treatments by MRS.
• Studies that used MRS that applied other nuclei such as phosphorus-31 (31P), fluorine-19 (19F), and carbon-13 (13C).
• Studies with irrelevant data.
• Studies with deficient results.
• Animal genetic-based studies, which compare expressed and non-expressed genes and the MRS findings, were limited.

### 2.5 Study risk of bias assessment

Two reviewers assessed the quality of each included study using the Robvis Visualization Assessment Tool (ROBINS-E Development Group (Higgins J, Morgan R, Rooney A, Taylor K, Thayer K, Silva R, Lemeris C, Akl A, Arroyave W, Bateson T, Berkman N, Demers P, Forastiere F, Glenn B, Hróbjartsson A, Kirrane E, LaKind J, Luben T, Lunn R, McAleenan A, McGuinness L, Meerpohl J, Mehta S, Nachman R, Obbagy J, O'Connor A, Radke E, Savović J, Schubauer-Berigan M, Schwingl P, Schunemann H, Shea B, Steenland K, Stewart T, Straif K, Tilling K, Verbeek V, Vermeulen R, Viswanathan M, Zahm S, Sterne J; Risk Of Bias In Non-randomized Studies - of Exposure (ROBINS-E). Launch version, June 20, 2023. Available from: https://www.riskofbias.info/welcome/robins-e-tool) for Observational Studies). A third reviewer resolved disagreements after two authors independently assessed the quality (Fig. 2).

## 3 Results

There were 1464 results identified from our selected databases; after removing 281 duplicates, 1183 abstracts and titles were observed, and 1142 articles were excluded; the 41 remaining articles were screened again, and 7 of them were removed since they reported results about treatment or maneuvering on less common types of MRS. After the final screening, nine studies were excluded, and eventually, 25 studies were included in this review (Fig. 1).

### 3.1 Characteristics of the studies

The population of the 25 included studies ranged from 4 to 1499 patients and healthy control groups (The overall sample size was more than 3833 people and 65 animals). The studies were conducted in Europe (10 studies: 2 in the UK, 2 in Italy, 3 in France, 2 in Germany, and 1 in Portugal), Asia (11 studies: 1 in Turkey, 1 in Taiwan, 6 in China, 1 in India, 1 in Egypt, and 1 in Japan), and North America (4 studies in the USA). As for the selected neurodegenerative diseases, 6 studies were included for AD, 4 for ataxia, 3 for HD, 4 for MS, 1 for MSAc, 5 for PD, 2 for PSP, and 3 for additional information.

### 3.2 Alzheimer

A study by Sultan et al. found much higher NAA/Cr ratios in MCI patients (1.74, 1.58, and 1.59) compared to AD patients (1.4, 1.5, and 1.79) in selected VOIs. In contrast, significantly higher mI/Cr and Cho/Cr ratios were observed in AD patients (1.51, 1.47, and 1.51) compared to MCI patients (1.1, 1.11, and 1.14) in the same selected regions [4].

Waragai et al. studied the NAA/Cr ratios in a group of patients and found that this ratio was lower in subjects who developed AD in seven years compared to cognitively healthy individuals (p < 0.0001). Similarly, mI/Cr ratios in those subjects were much higher than in subjects who did not develop AD (p = 0.046), and this trend was still present after seven years. Besides, MCI and AD patients had much lower NAA/MI ratios (p < 0.0001 for both) than healthy individuals [16].

Mullins et al. studied levels of essential metabolites in AD patients and two groups of normal patients with different age ranges (Table 1). A significant difference was observed in Glc levels between groups (F [2, 73] = 8.752, P = 0.0004), as Glc was higher in AD patients than in the two normal groups (P = 0.0003) (P = 0.001). The same trend was observed for Lactate (Lac) and ascorbate (Asc). The difference in other substances (Gln, Glu, PCh, and Scy) was observed solely compared to younger normal individuals: Scy was higher in AD patients and older normal subjects (P = 0.007 and P = 0.016, respectively) (F [2, 55] = 4.241, P = 0.019), Glu had lower levels in AD patients (P = 0.00008) and older normal subjects (P = 0.004) (F[2,75] = 9.071, P = 0.000), and Gln was higher in AD patients compared to younger normal subjects (P = 0.002) [17].

**Table 1:** Summary of articles related to Alzheimer disease.

Authors	Type of study	Year	Region	Age of patients	Sample size	Magnetic Tesla	Imaging method	Voxel/Matrix size	VOIs	Main findings
Amina Ahmed Sultan MD et al.	Original research study	2017	Egypt	50-73 (mean: 61.6)	13	1.5T Siemens Magnetom and Signa HDe GE Healthcare	Multi-voxel MR spectroscopy	–	hippocampal, temporal and parietal	NAA/Cr ratio • in hippocampus: 1.41 ± 0.21 • in temporal: 1.05 ± 0.15 • in parietal:1.79 ± 0.08 • mI/Cr ratio • in hippocampus: 1.51 ± 0.15 • in temporal: 1.47 ± 0.13 • in parietal: 1.51 ± 0.13 • Cho/Cr ratio • in hippocampus: 1.27 ± 0.8 • in temporal: 1.38 ± 0.17 • in parietal: 1.36 ± 0.08
Masaaki Waragai et al.	Longitudinal cohort study.	2017	Japan	mean age: 74.8 ± 5.2	289(21 of them developed AD after 7 years of follow-up	1.5T Vantage Titan Toshiba Medical Systems	Single-voxel method with a spin echo sequence.	8 cm3 (2 × 2 × 2 cm)	Posterior Cingulate Cortex	• The NAA/Cr: lower in AD • The MI/Cr: higher in progressor AD • The NAA/MI lower in AD.
Roger Mullins et al.	Original cross-sectional study	2018	USA	over 60 (older CN > 60 and younger CN < 60)	25 participants with high probability AD	3T Philips Achieva	A single voxel technique	25 × 18 × 20 mm	posterior cingulate/precuneus	• Glc: higher in AD patients than older and younger CNs. • Lactate (Lac) and ascorbate (Asc): higher in AD than younger and older CNs. • Gln: higher in AD group than the younger CNs • Glu: lower in AD and older CN than the younger CN • PCh: higher in younger CN than the older CN and AD • Scy: higher in AD older CN than the younger CN
Micaela Mitolo et al.	Case-control study	2019	Italy	70.8 ± 9.3 y AD 73.9 ± 7.4 y MCI	23 AD 38 MCI	1.5T GE-HDx	Single voxel 1H spectra	2.0 × 2.0 × 2.0 cm	Posterior Cingulate Cortex	• NAA/mI ratio • in CN: (mean 1.76 ± 0.17) • in MCI:(mean 1.56 ± 0.38) • in AD (mean 1.32 ± 0.25) • in baseline MCI converters: (mean 1.42 ± 0.23) • in MCI that did not develop AD: (mean 1.85 ± 0.47)
Małgorzata Marjanska et al.	Longitudinal cohort study	2019	USA	78 ± 7	16	7T Siemens MAGNETOM	Single-shot spectra	–	posterior cingulate cortex (PCC) And occipital cortex (OCC)	• Asc, mIns, and tCho: higher in AD. • PE: lower in PCC of participants with AD. • NAAG: lower in OCC of participants with AD.
Qianyun Chen et al.	Retrospective study	2022	China	50–80	16 early AD 15 late AD	3T Philips Achieva	Point-resolved spectroscopy(PRESS)	8 cm3 (2 × 2 × 2 cm)	Lower posterior cingulate	• NAA/Cr: lower in early and late AD. • ml/Cr: higher in late AD.

Mitolo et al. proposed differences in NAA/mI ratios in the posterior cingulate cortex (PCC) as the primary factor in differentiating MCI (mean 1.56 ± 0.38) (p = 0.011) and AD (mean 1.32 ± 0.25) (p = 0.038) patients from healthy older individuals (mean 1.76 ± 0.17) [18].

Marjanska et al. concluded that Asc was the primary biomarker in distinguishing between AD patients and cognitively normal individuals. They also observed much higher MIns and tCho levels in the PCC of patients with AD (p ≤ 0.004) but lower levels of PE. AD patients had lower NAAG levels in their occipital cortex (OCC) but higher Asp and mIns [19].

**Fig. 3:**
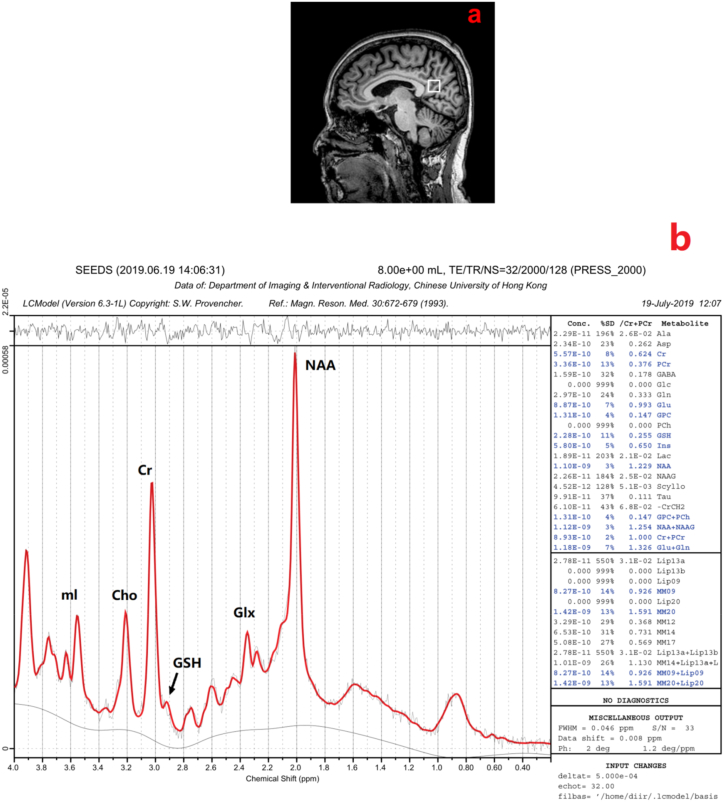
(a) Example of the 1H-MRS voxel localization. (b) MR proton spectra. mI, myoInositol; Cho, cholinecontainingcompound; Cr,creatine; GSH,glutathione; Glx,glutamate and glutamine; NAA,N-acetylaspartate; ppm, parts per million [20].. (Adapted with permission.)

Chen et al. investigated metabolite levels in two patient groups with early and late-stage AD. The group with early AD had lower NAA/Cr (p = 0.003). Those with late AD had higher ml/Cr (p = 0.04) and lower NAA/Cr (P = 0.002) levels. A significant difference was observed in the levels of these metabolites between the two AD groups and cognitively normal individuals (As shown in Fig. 3(A and B)). [20].

### 3.3 Progressive supranuclear palsy

In the study of Barbagallo et al., levels of Cho, Glx, GSH, and mI showed no difference in PSP patients and cognitively normal individuals. However, subjects with PSP-RS (progressive supranuclear palsy-Richardson syndrome) had significantly lower levels of NAA, Cr, and Scyllo, with FDR-corrected P-values of 0.02, 0.02, and 0.01, respectively. Only the Scyllo/Cr (FDR-corrected P-value = 0.02) ratio was decreased in PSP-RS patients [13] (Table 2).

**Table 2:** Articles summaries included Progressive supranuclear palsy.

Authors	Type of study	Year	Region	Age of patients	Sample size	Magnetic Tesla	Imaging method	Voxel/Matrix size	VOIs	Main findings
Gaetano Barbagallo et al.	Observational cross-sectional study	2019	Italy	PSP-RS mean:63.6 CN mean: 66.2	16 (PSP-RS) 18 (CN)	3T Siemens	Single- voxel 1H-MRS	25 × 25 × 15 mm	right and left supplementary motor area (SMA)	• No significant differences in Cho, Glx, GSH, and mI between groups. • NAA, Cr, and Scyllo lower in PSP-RS • Scyllo/Cr: the only ratio reduced in PSP-RS
Alexander G. Murley et al.	Longitudinal cohort study	2021	UK	mean: 66.2	44	7T Siemens MAGNETOM	Single-voxel technique	2 × 2 × 2 cm^3^	Right inferior frontal gyrus and occipital lobe	GABA and Glutamate: lower in PSP.

**Fig. 4:**
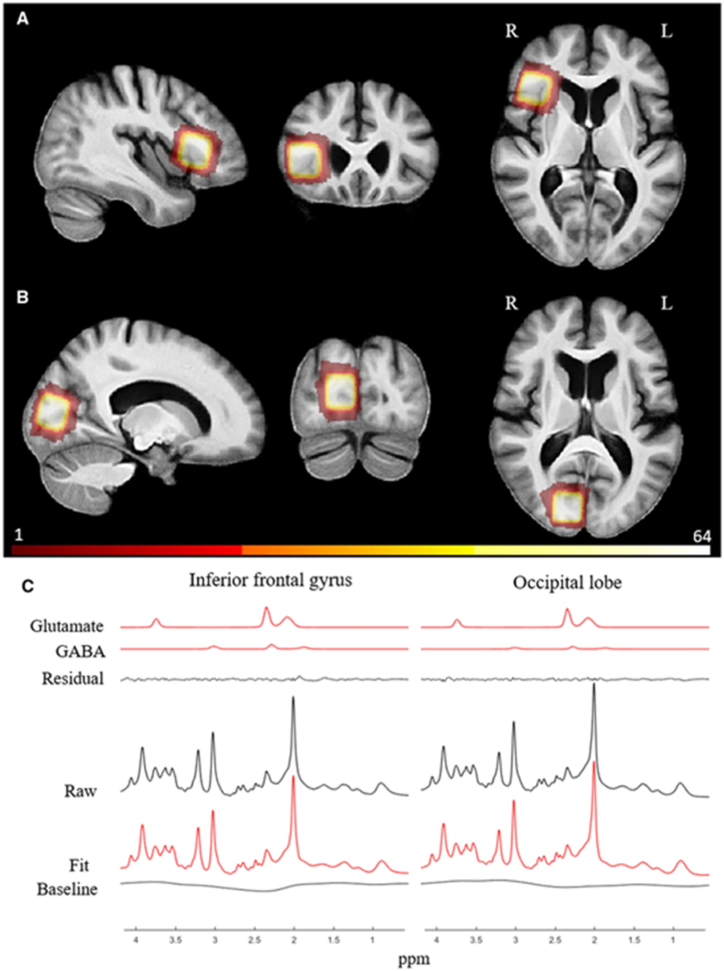
Spectroscopy voxel composition and location. (A) The frontal voxel (sum of all subjects) superimposed on a mean structural image of all participants. (B) Occipital's VOI. (C) Mean spectra for all subjects, including raw data, LCModel fit, baseline, residual (fit - raw data), glutamate, and GABA fits [21]. (Adapted with permission.).

Murley et al. measured glutamate and GABA concentrations in the right inferior frontal gyrus and occipital lobe (Fig. 4(A-C)). GABA and glutamate in the right inferior frontal gyrus were decreased in PSP compared to controls [21]. (Table 2).

### 3.4 Multiple sclerosis

Duan et al. reported a significant decrease in NAA/Cr and NAA/Cho ratios (mean 1.95 versus 2.15, P = 0.001; mean 1.85 versus 2.01, P = 0.039; 1.54 versus 1.85, P = 0.000, respectively). In MS patients, Cho was also higher than that of normal controls (mean 1.29 versus 1.17, P = 0.031) [22].

Yarraguntla et al. assessed 48 patients with RRMS. The subjects were divided into three groups according to the Fatigue Severity Scale (FSS): low fatigue (LF, FSS ≤3), moderate fatigue (MF, FSS = 3.1–5), and high fatigue (HF, FSS ≥5.1). The LF group had much higher NAA + NAAG)/(Cr + PCr) ratios than the HF subjects (P = 0.018) in the right anterior quadrant (RAQ). Similar results were found in the left anterior quadrant (LAQ), so the LF patients had much higher (NAA + NAAG)/(Cr + PCr) ratios than patients in the HF group (P = 0.02) [23].

Swanberg et al. reviewed the CNS biomarker levels in patients with MS and MS subtypes in in-vivo1H-MRS results. Creatine-referenced N-acetyl aspartate and N-acetyl aspartate relative to non-creatine references like water or phantom acquisitions showed reductions compared to control in mixed or unspecified MS lesions, white matter, normal-appearing white matter, and mixed tissue in RRMS, white matter, normal-appearing white matter, gray matter, mixed tissue, and spine; and in PMS lesions, white matter, normal-appearing white matter, gray matter, and mixed tissue. The fluctuations of Cr remained unclear, but it showed increases in MS lesions. Creatine-referenced inositol or myo-inositol increased in mixed or unspecified MS lesions; in relapsing-remitting lesions, white matter with normal appearance, and mixed tissue; and in progressive mixed tissue and spine. Other inositols showed higher levels in MS lesions with white matter with normal appearance and gray matter; in RRMS lesions, white matter with normal appearance and mixed tissue; and in progressive lesions, white matter with normal appearance, gray matter, and mixed tissue.

**Fig. 5:**
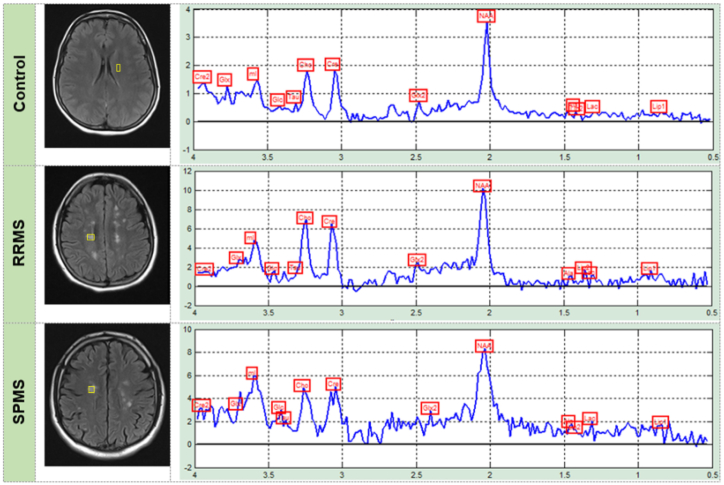
MR images and MRS signals of healthy controls and RRMS and SPMS patients. The NAA peak in progressive MS cases has a tendency to decrease, however, Cr and Co show and increasing trend in MS patients [11].. (Adapted with permission.)

There were decreased levels of non-creatine-referenced glutamate in MS and relapsing-remitting mixed tissue. However, in MS lesions and white matter with normal appearance, the levels were decreased. The final levels of glutathione were decreased in gray matter voxels measured superior to the ventricles but not those of the white matter. Findings indicated lower levels of glutathione in secondary progressive mixed-tissue voxels in the frontal and parietal cortex [2] (Table 3). Ziya EKŞİ et al. analyzed the peaks of biomarkers. Based on their study, the mean level of NAA peaks was 5.93 ± 2.92, 9.24 ± 2.01 in healthy controls, and 7.70 ± 2.85 in RRMS patients and SPMS (secondary progressive MS) patients. These findings indicate that the NAA peak in progressive MS cases has a tendency to decrease (Fig. 5). Cr and Cho had an increasing trend in MS patients, and the mean levels of Cr and Cho in the control group were 2.93 ± 1.75 and 2.83 ± 1.86, respectively. In RRMS patients, the mean Cr level was 5.88 ± 1.41, and the mean Cho level was 5.89 ± 1. These amounts were 4.93 ± 1.95 and 4.93 ± 2.11 in SPMS patients, respectively [11] (Table 3).

**Table 3:** Summary of articles related to Multiple Sclerosis. RRMS: relapsing-remitting multiple sclerosis; SPMS: secondary progressive multiple sclerosis; CN: control group.

Authors	Type of study	Year	Region	Age of patients	Sample size	Magnetic Tesla	Imaging method	Voxel/Matrix size	VOIs	Main findings
Yunyun Duan et al.	Randomized controlled trial	2017	China	PRMS mean age (37.8) CN mean age (33)	24(CN) 24(PRMS)	1.5T Magnetom Sonata	Point resolved spectroscopy (PRESS)	1.0 × 1.0 × 1.0 cm^3^	normal appearing white matter (NAWM)	NAA,NAA/Cr and NAA/Cho: lower in MS. Ch: Higher in MS
Kalyan Yarraguntla et al.	Observational longitudinal study	2019	USA	Female 41 (±2.4) Male 39 (±2.3)	48 (PRMS)	3T Siemens Verio MR scanner	Multivoxel point resolved spectroscopy (PRESS)	10 × 10 × 15 mm	left anterior quadrant (LAQ) and right anterior quadrant (RAQ)	(NAA + NAAG)/(Cr + PCr): higher in the LF group compared with the HF group in RAQ. (NAA + NAAG)/(Cr + PCr): higher in the LF group compared with the HF group in LAQ
**Review Article**										
Kelley M. Swanberg et al.	Review	2019	USA	26 studies					multiple sclerosis lesions, white matter, normal appearing white matter, gray matter, mixed tissue, and spine.	Creatine: increase in mixed or unspecified multiple sclerosis lesions. Creatine referenced inositol or myoinositol: increase in mixed or unspecified multiple sclerosis lesions. Glutamate not referenced to creatine: decrease in multiple sclerosis mixed tissue and relapsing-remitting mixed tissue and Increase in multiple sclerosis lesions and normal-appearing white matter. glutathione: decrease in gray but not white matter, secondary progressive mixed-tissue voxels in the frontal and parietal cortex. N-acetylaspartate: reduce.
Ziya EKŞİ et al.	Observational and Cross-sectional study	2020	Turkey	34.2 ± 8.85 (PRMS) 48.1 ± 8.84 (SPMS)	36 (PRMS) 25(SPMS)	1.5T Siemens Avanto	Single-voxel technique	–	MS brain lesions and inside spinal plaques	NAA peaks: mean 9.24 ± 2.01, and 7.70 ± 2.85 in RRMS patients, and SPMS patients. Cr and Cho: mean 5.88 ± 1.41 and 5.89 ± 1.42, in RRMS patients and 4.93 ± 1.95 and 4.93 ± 2.11, in SPMS patients. Decreasing trend in NAA peak in progressive forms of MS and an increasing trend in Cr an Co peaks in MS patients.

### 3.5 Parkinson's disease

Guan et al. reported lower NAA/Cr and NAA/Cho ratios in substantia nigra (1.80 (1.40–2.10) and 2.02 (1.74–2.74), respectively), globus pallidus (1.65 (1.44–1.93) and 1.89 (1.49–2.45)), prefrontal lobe (1.52 (1.23–1.89) and 1.76 (1.41–2.28)), hippocampus (1.68 (1.48–1.92) and 2.26 (1.73–2.66)), cuneus gyrus (1.69 (1.53–1.88) and 2.27 (1.93–3.12)), dorsal thalamus (1.85 (1.59–2.07) and 1.96 (1.47–2.44)) in total PDs in compare to controls. Furthermore, NAA/Cr and NAA/Cho were lower in SN (1.75 (1.41–2.28) and 1.98 (1.69–2.72)), GP (1.69 (1.43–2.12) and 1.87 (1.36–2.40)), PL (1.64 (1.30–2.06) and 1.67 (1.46–2.39)), HI (1.77 (1.52–2.13) and 2.07 (1.52–2.73)), CG (1.80 (1.39–2.07) and 2.06 (1.76–2.75)), DT (1.80 (1.54–1.99) and 1.84 (1.46–2.21)) in unilateral impairment; as well as bilateral in SN (1.84 (1.40–2.01) and 2.52 (1.77–2.86)), GP (1.60 (1.41–1.85) and 1.60 (1.41–1.85)), PL (1.64 (1.30–2.06) and 1.67 (1.46–2.39)), HI (1.77 (1.52–2.13) and 2.07 (1.52–2.73)), CG (1.80 (1.39–2.07) and 2.06 (1.76–2.75)), DT (1.80 (1.54–1.99) and 1.84 (1.46–2.21)). Lower amounts of NAA/Cr in SN (1.40 (1.27–2.19)), GP (1.66 (1.23–2.21)), HI (1.77 (1.43–2.21)), DT (1.74 (1.45–2.17)) were observed in cognitively normal subjects and patients with mild impairment, but there was a reduction in NAA/Cho in GP (1.73 (1.32–2.67)), CG (2.29 (1.89–3.39)), and DT (1.63 (1.30–2.37)). Cognitively impaired patients had lower NAA/Cr and NAA/Ch rations in all of the six regions: SN (1.85 (1.43–2.14) and 2.02 (1.69–2.82), respectively), GP (1.65 (1.45–1.91) and 1.91 (1.42–2.53)), PL (1.52 (1.21–1.86) and 1.67 (1.28–2.14)), HI (1.66 (1.48–1.91) and 2.20 (1.75–2.65)), CG (1.68(1.51–1.85) and 2.27 (1.92–3.06)), DT (1.87 (1.61–2.09) and 2.12 (1.51–2.45)). Cho/Cr ratios were higher for all PDs in CG (0.76 (0.56–1.01)) and DT (0.99 (0.78–1.32)) [24].

Cao et al. examined on medulla oblongata (−0.081 ± 0.082), substantia nigra (−0.27 ± 0.059), putamen (0.083 ± 0.0750), and motor cortex (0.043 ± 0.069) and found that the ratio of NAA/Cr was lower in PDs in SN, unlike other VOIs in which there was no significant difference. There was no difference in Cho/Cr ratios in any of the regions [25].

**Fig. 6:**
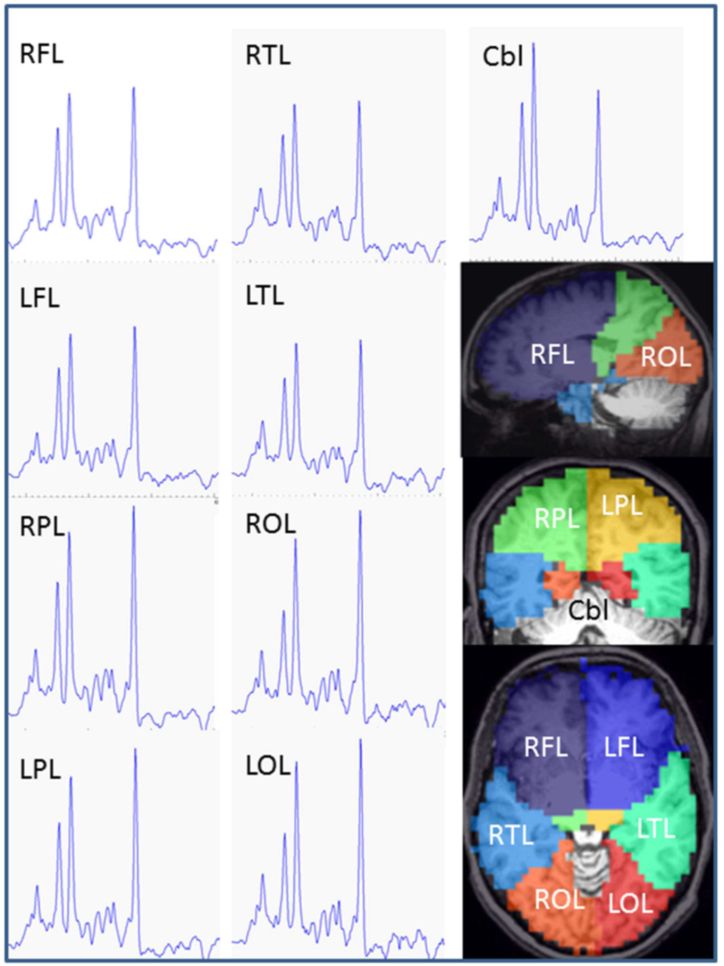
An illustration of MR spectra of the brain lobes and cerebellum belonging to a female PD patient. The brain areas are approximately corresponded to the brain lobes in the present study. Frontal lobe: BA 4, 6, 8, 9, 10, 11,1 2, 24, 25, 32, 33, 44, 45, 46, 47, head of caudate nucleus, accumbens, anterior part of putamen and pallidum, anterior cingulum. Parietal lobe: BA 1, 2, 3, 5, 7, 23, 31, 39, 40, thalamus, subthalamic nucleus, posterior cingulum. Temporal lobe: BA 13, 14, 15, 16, 20, 21, 22, 26, 27, 28, 29, 30, 34, 35, 36, 37, 38, 41, 42, 43, hippocampus, posterior parts of putamen and pallidum, caudatus tail, amygdala. Occipital lobe: BA 17, 18, 19. RFL, right frontal lobe; LFL, left frontal lobe; RPL, right parietal lobe; LPL, left parietal lobe; RTL, right temporal lobe; LTL left temporal lobe; ROL right occipital lobe; LOL left occipital lobe; Cbl, cerebellum; BA, Brodmann area [26].. (Adapted with permission.)

Klietz et al. researched the early stages of PD. The results are as follows: in PDs, NAA decreased significantly in the right temporal lobe (mean: 8.0), right parietal lobe (mean: 9.94), and right occipital lobe (mean: 9.61), with a slight decrease in both frontal lobes (mean: right 9.71 and left 9.69). Cho just showed a slight reduction in the right temporal lobe (mean: 1.8). Lower Cho amounts were observed in the right temporal lobe (mean: 7.78), but its amounts in the right parietal lobe were only slightly reduced (mean: 8.59). Glutamate significantly decreased in the right temporal lobe (mean: 7.05) and right occipital lobe (mean: 7.56). Glutamine only showed a slight increase in the left temporal lobe (mean: 3.34), and in the end, there were no significant differences in mIns between PDs and controls (Fig. 6) [26].

A review by Gu et al. gathered all data on the early diagnosis of PD from proton MRS in papers published in 1997–2018. They reported that NAA/Cr ratios were lower in PDs compared to controls in substantia nigra or globus pallidus [10].

In the study of Huang et al. on monkeys, the results showed that GABA concentration in the affected side (before PD: 0.356 ± 0.123, after PD: 0.405 ± 0.161) of the striatum was higher than its healthy side (before PD: 0.266 ± 0.114, after PD: 0.323 ± 0.947) [27].

### 3.6 Huntington's disease

**Fig. 7:**
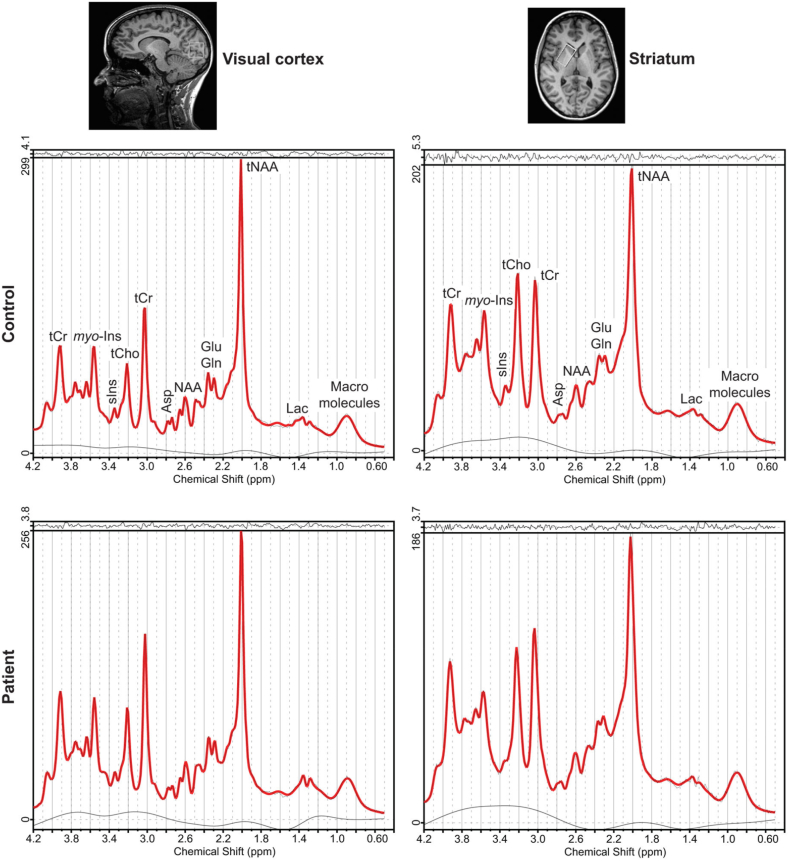
Voxel positioning, spectra quality and model fitting by LCModel in the visual cortex and striatum. Spectra were acquired in an acquisition voxel of 25 × 25 × 25 mm3 in the visual cortex and 34 × 19 × 23 mm3 in the striatum using the modified semi-LASER sequence (TR = 5000 ms, TE = 28 ms, averages = 64). The black lines are the raw spectra, whilst the red lines are the LCModel fits. Asp: aspartate; Gln: glutamine; Glu: glutamate; Lac: lactate; myo-Ins: myo-inositol; NAA: N-acetylaspartate; sIns: scyllo-inositol; tCho: total choline; tCr: total creatine; tNAA: total N-acetylaspartate [9].. (Adapted with permission.).

Adanyeguh et al. (2018) researched the visual cortex and striatum, and the results showed a 7 % increase in tCr in the visual cortex in HDs and no difference in other neurometabolites. In the striatum, HDs displayed a 20 % decrease in Glu and a 12 % decrease in tCr; moreover, tNAA showed a 4 % tendency to decrease. Ascorbic acid, scyllo-inositol, lactate, and taurine in a 50 % increased threshold showed no significant differences between HDs and controls (Fig. 7). The findings of Adanyeguh et al. (2021) on corpus callosum indicated much lower tNAA amounts in the corpus callosum of HDs, whereas there was no difference in tCho [9,28].

Pépin et al. examined mouse models and found that tNAA, Tau, Glu, and tCho decreased in contrast with Gln, which increased in the striatum [1]. (Table 5).

**Table 4:** Summary of articles related to Parkinson disease. PD: Parkinson disease; CN: control group; SN: substantia nigra; GP: globus pallidus; PL: prefrontal lobe; HI: hippocampus; CG: cuneus gyrus; DT: dorsal thalamus; MO: medulla oblongata; PU: putamen; MC: motor cortex; RFL: right frontal lobe; LFL: left frontal lobe.

Authors	Type of study	Year	Region	Age of patients	Sample size	Magnetic Tesla	Imaging method	Voxel size	VOIs	Main findings
Jitian Guan et al.	Observational and Cross-sectional study	2017	China	61.6 ± 6.4	42(PD) 20(CN)	3T Sigma HDx Twin speed	Point resolved spectroscopy (PRESS)	8 × 10 × 1 cm	SN,GP,PL,HI,CG and DT on both sides of the brain	NAA/Cho & NAA/Cr: Lower in 6 regions (PDs,Uni & Bilaterals) NAA/Cr: Lower in SN,GP,HI,DT regions (Mild & No Cognitive) Cho/Cr: Higher in CG,DT regions (PDs)
Hongmei Cao et al.	Observational and Cross-sectional study	2017	China	60.5 ± 1.3 vs 57.4 ± 1.3	22(PD) 15(CN)	1.5T Philips Gyroscan Intera	Single-voxel technique	10 × 10 × 15 mm^3^	MO,SN,PU,MC	NAA/Cr: Lower in SN (PDs) Cho/Cr: no difference in any region(PDs)
Martin Klietz et al.	Original research study	2019	Germany	60.2 ± 7.2	20(PD) 20(CN	3T Siemens Verio MR scanner	Multi-voxel technique	5.6 × 5.6 × 10 mm^3^	RFL,LFL,RTL,LTL,RPL,LPL,ROL,LOL,Cbl	NAA: RTL(-8.6 %) RPL(-6.7 %) ROL(-8%) Glu: RTL(-9.9 %) ROL(-10.2 %) Gln: LTL(20 %)
**Systematic Review**										
Wenbin Gu et al.	Review	2022	China	16 studies					SN,GP	NAA/Cr: Lower in GP and SN regions (early stage PD)
**Monkey models**										
Lixuan Huang et al.	Original research study	2019	China	6–9 years	8	3T Siemens	Image selected in vivo spectroscopy (ISIS)	20 mm × 20 mm × 20 mm	striatum	GABAconcentration increased in injured side of striatum of PD monkeys

**Table 5:** Summary of three articles related to Huntington disease. HD: Huntington disease; CN: control group.

Authors	Type of study	Year	Region	Age of patients	Sample size	Magnetic Tesla	Imaging method	Voxel size	VOIs	Main findings
Isaac M. Adanyeguh et al.	Original research study	2018	France	45.6 ± 12.7	10 (HD) 10(CN)	3T Siemens Magnetom Trio scanner	Semi-LASER	25 × 25 × 25 mm^3^	Striatum & visual cortex	Cr: Higher in visual cortex(HDs) Glu & Cr: Lower in striatum(HDs)
Isaac M. Adanyeguh et al.	Observational clinical study	2021	France	45.5 ± 6.8	20(HD) 20(CN)	3T Siemens Magnetom Prisma scanner	Semi-LASER	15 × 32 × 8 mm^3^	corpus callosum	tNAA: Lower in corpus callosum(HDs)
**Mouse models**										
Jérémy Pépinet et al.	Preclinical study	2020	France	12 month	–	11.7T Bruker	Semi-LASER	2 × 2 × 2 mm^3^	corpus callosum	tNAA: Lower in left striatum (−17.4 % Ki140Hetero & −24.3 % Ki140Homo & −36.4 % R6/1Hetero) Tau: decrease (−25.9 % Ki140Hetero & −33.3 % Ki140Homo) Glu: decrease (−13.0 % Ki140Hetero & −14.9 % Ki140Homo) Gln: Increase (+27.4 % Ki140Homo) tCho: decrease (−21.7 % Ki140Homo)

### 3.7 Ataxia and motor system atrophy

Krahe et al. investigated SCA and FA, and their findings are discussed below:

NAA/Cr: This ratio was much lower in the cerebellum of SCA2 patients than in SCA3, SCA6, and FA patients (U = 47.000, z = −3.749; U = 24.00, z = −3772; and U = 1.000, z = −3.502, respectively).

Cho/Cr: The cerebellum of SCA2 patients had lower Cho\Cr ratios than other ataxia subtypes, including SCA1, SCA3, SCA6, and FA (U = 9.500, z = −3635; U = 18.000, z = −4.033, U = 1.000, z = −4.540; and U = 126.000, z = −2.751, respectively). This ratio was also lower in the cerebellum of SCA1 patients than those with SCA6 patients (U = 15.000, z = −3.223).

MI/Cr: A difference was observed in the mI/Cr ratio in the cerebellum of SCA2 patients compared to SCA1 and SCA3 subjects (U = 3.000, z = −2.893; U = 0.000, z = −3.000, respectively) as that of SCA2 was higher. Other metabolites had no significant difference in the cerebellum and pons of patients with varying genotypes of ataxia [6] (Table 6).

**Table 6:** Summary of articles related to ataxia diseases.

Authors	Type of study	Year	Region	Age of patients	Sample size	Magnetic Tesla	Imaging method	Voxel size	VOIs	Main findings
**Systematic Review**										
Janna Krahe et al.	Review	2020	Germany	1499 patients in total: SCA1 = 223, SCA2 = 298, SCA3 = 711, SCA6 = 165, and FA = 102					cerebellum, pons,GM,BG,CC,PLWM	● NAA/Cr: decrease (SCA 1,2,3,6 & FA) ● Cho/Cr: decrease (SCA 1,2,3) ● Ml/Cr: higher in cerebellum (SCA2) ● Cho/Cr: higher in cerebellum (SCA6)
Hung Chieh Chen et al.	Retrospective observational study	2022	Taiwan	41.4 ± 12.66	398	1.5T Signa EXCITE	Stimulated echo acquisition mode (STEAM)	2 cm × 2 cm × 2 cm	cerebellum, vermis	● 1 year duration: ● NAA/Cr: (0.94 ± 0.14 in CE) & (0.86 ± 0.09 in VE) ● Cho/Cr: (0.74 ± 0.17 in CE) & (0.69 ± 0.09 in VE) ● 2–3 years duration: ● NAA/Cr: (0.88 ± 0.19 in CE) & (0.81 ± 0.11 in VE) ● Cho/Cr: (0.70 ± 0.13 in CE) & (0.69 ± 0.10 in VE) ● 4–5 years duration: ● NAA/Cr: (0.85 ± 0.15 in CE) & (0.82 ± 0.09 in VE) ● Cho/Cr: (0.68 ± 0.11 in CE) & (0.69 ± 0.08 in VE) ● 6–8 years duration: ● NAA/Cr: (0.83 ± 0.18 in CE) & (0.81 ± 0.09 in VE) ● Cho/Cr: (0.68 ± 0.11 in CE) & (0.68 ± 0.10 in VE) ● Longer than 8 years duration: ● NAA/Cr: (0.78 ± 0.15 in CE) & (0.76 ± 0.13 in VE) ● Cho/Cr: (0.65 ± 0.11 in CE) & (0.65 ± 0.10 in VE)
Vishwa Rawat et al.	Original research study	2022	India	40–65	6	3T Philips	Point-resolved spin-echo pulse sequence (PRESS)	15 mm × 15 mm × 15 mm	cerebellum, vermis	● tNAA & tCho: Lower in vermis (GAs) ● tNAA: Lower in right cerebellum (GAs) PCh: higher in younger CN than the older CN and AD ● Scy: higher in AD older CN than the younger CN
**Human and Animal models**										
Catarina Oliveira Miranda et al.	Preclinical research study	2022	Portugal	4 human (2 MJD patients 33 and 57 years old and 2 controls) 57 animals of 2, 4 or 16 months old		3T Siemens Magnetom TIM Trio 3 Tesla scanner/9.5T Bruker	Point-resolved spin-echo pulse sequence (PRESS)	1 × 1 × 1 mm (Human) 4.0 mm × 1.1 mm × 1.0 mm (Animal)	cerebellum	● tNAA &NAA & Glu & Tau: lower in cerebellum(in MJDs) ● Ins: higher in cerebellum (in MJDs) ● NAA/Ins & NAA/tCho: lower in cerebellum(in MJDs)

**Fig. 8:**
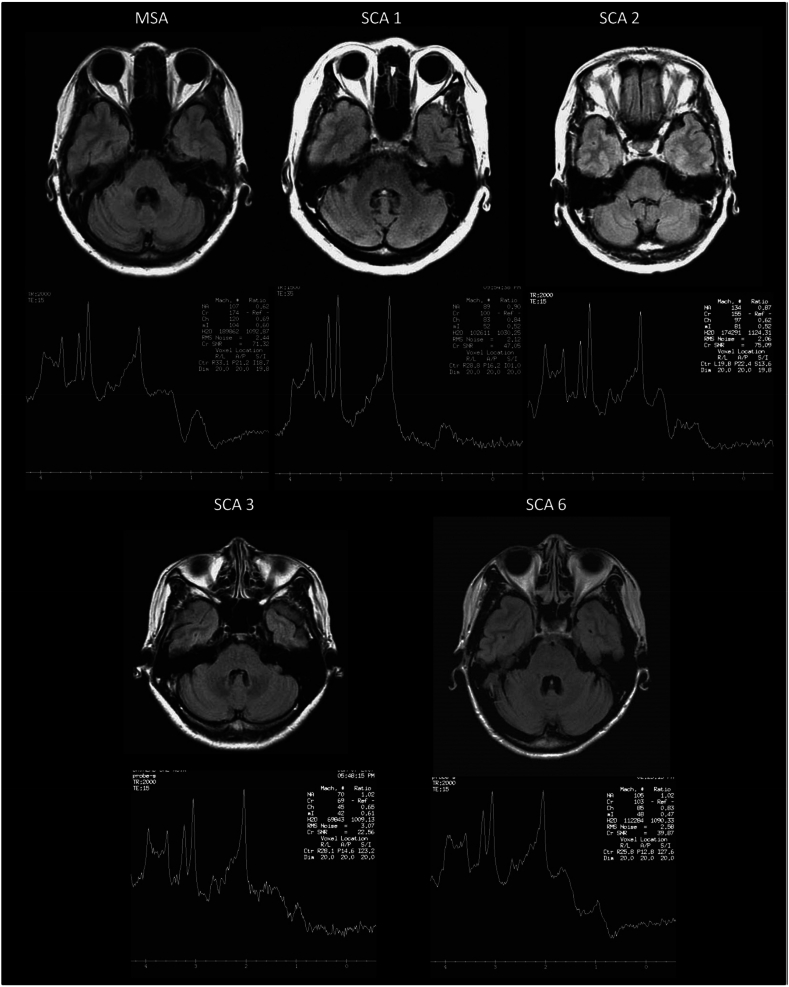
Axial FLAIR images at pons level and cerebellar MRS of patients with MSA or SCAs [5]. (Adapted with permission).

Chen et al. found a significant reduction in NAA/Cr and Cho/Cr ratios in cerebellar and vermis VOIs during 1–8 years of SCA and MSA-c [5] (Fig. 8).

In the study of Rawat et al. on GA, there were lower N-acetyl aspartate (tNAA: N-acetylaspartate + N-acetylaspartate glutamate) and choline (tCho: glycerylphosphorylcholine + phophocholine) amounts in the vermis and right cerebellum [8].

Miranda et al. studied human and animal models. Both models showed similar structural and anatomical cerebellar alteration. The results indicated that tNAA (0.91 ± 0.27 vs. 1.24 ± 0.26), Glu (4.29 ± 1.25 vs 5.80 ± 1.27), and Tau (4.35 ± 1.22 vs. 5.73 ± 1.66) were decreased whereas myo-inositol (6.97 ± 2.32 vs. 4.95 ± 1.25) was increased in cerebellum. Moreover, some clinical ratios such as NAA/Ins (0.48 ± 0.15 vs. 1.04 ± 031) and NAA/tCho (o: 2.22 ± 0.39 vs. 3.65 ± 0.50) were decreased [7] (Table 6).

Kadodwala et al. worked on MSA-c and found that NAA/Cr was lower in the vermis (mean 0.67) and cerebellar hemisphere (mean 0.72). The Cho/Cr ratio also was lower in the vermis (mean: 0.62) but not in the cerebellar hemisphere (mean: 0.07) [12] (Table 7).

**Table 7:** Summary of articles related to motor system atrophy.

Authors	Type of study	Year	Region	Age of patients	Sample size	Magnetic Tesla	Imaging method	Imaging method	VOIs	Main findings
Viren H. Kadodwala et al.	Retrospective case-control study	2019	UK	58.7 ± 6.6	20	3T Philips ACHIEVA	Point-resolved spectroscopy (PRESS)	2.0 × 1.0 × 2.0 cm^3^	superior cerebellar vermis and the deep cerebellar white matter of the right hemisphere	NAA/Cr & Cho/Cr: decrease in vermis (MSA-c) NAA/Cr: decrease in cerebellar hemisphere (MSA-c)
Hung-Chieh Chen et al.	Retrospective observational study	2022	Taiwan	55.72 ± 7.71	286	1.5T Signa EXCITE	Stimulated echo acquisition mode (STEAM)	2 cm × 2 cm × 2 cm	cerebellum, vermis	1 year duration: NAA/Cr: (0.66 ± 0.12 in CE) & (0.73 ± 0.08 in VE) Cho/Cr: (0.60 ± 0.10 in CE) & (0.61 ± 0.07 in VE) 2–3 years duration: NAA/Cr: (0.65 ± 0.11 in CE) & (0.73 ± 0.08 in VE) Cho/Cr: (0.59 ± 0.10 in CE) & (0.60 ± 0.08 in VE) 4–5 years duration: NAA/Cr: (0.59 ± 0.11 in CE) & (0.64 ± 0.10 in VE) Cho/Cr: (0.54 ± 0.13 in CE) & (0.57 ± 0.09 in VE) 6–8 years duration: NAA/Cr: (0.56 ± 0.13 in CE) & (0.62 ± 0.09 in VE) Cho/Cr: (0.48 ± 0.11 in CE) & (0.52 ± 0.10 in VE) Longer than 8 years duration: NAA/Cr: (0.54 ± 0.12 in CE) & (0.63 ± 0.10 in VE) Cho/Cr: (0.47 ± 0.11 in CE) & (0.52 ± 0.09 in VE)

## 4 Discussion

This systematic review intended to collect the latest outcomes relevant to biomarker changes for each neurodegenerative disease; hence, radiologists and physicians can have better access to a comprehensive and accurate database. No other reviews were published with the same content when this study was being conducted. We searched four electronic databases; the search yielded 1464 studies, 25 of which were included in this systematic review after a two-step screening process.

The included studies provided us with sufficient reports about the selected diseases. Six studies evaluated AD, NAA, and its ratios to other biomarkers were measured in all selected studies, NAA/Cr [4,16,20] (Fig. 3) NAA/MI generally decreased in AD patients in their related VOIs [16,18], although MI/Cr was higher [4,16,20]. In addition, Asc is an identifiable biomarker that is elevated in individuals with Alzheimer's disease, as are Glc and Lac, although their significance is comparatively lower [17,19]. A few studies also analyzed the mean levels of these biomarkers in different areas (Table 1).

We could find only two studies about PSP, one of which measured NAA, Cr, Scyllo, and Scyllo/Cr in PSP-RS, and all were reduced (Fig. 4). The mechanism of the effect of Scyllo reduction in PSP patients remains uncertain. Probable reasons include lower blood-brain transport or a reduction in brain biosynthesis [13,21] found that PSP patients had lower GABA and Glu levels (Table 2).

Four of the selected articles referred to MS even though they used different methods in their evaluations. Moreover, it is necessary to consider NAA as a metabolite in distinguishing the type of MS [11]. In PRMS, NAA amount and ratios decrease [11,22], but another study divided PRMS patients into three groups of LF, MF, and HF, and the results were different between LF and HF groups [23]. Another type of MS discussed in studies was SPMS, which has increasing Cr and Co levels [11] (Fig. 5). Finally, one of the articles collected an informative database about MS and all the common subtypes [2] (Table 3).

Five studies reported statics of effective biomarkers in PD; NAA/Cr ratios dropped in PD patients. The NAA had a comparable decline in patients with Parkinson's disease; however, the decrease was only observable in specific regions of the brain [26]. The findings indicate the biomarker role of these metabolites in the early diagnosis of PD and cognitive impairment in PD patients [10,24,and25]]. There were some contrasting results about Cho/Cr high levels in CG and DT, but there were no differences in this ratio in MO, SN, PU, and MC [24,25]. Another study examined GABA levels in monkey models, which increased in the striatum of PD monkeys [27] (Table 4).

Three of the articles reported data about HD, including one that used mouse models that resulted in a reduction of Glu, tCHO, Tau, and tNAA [1], which was also lower in human models [28]. Cr was higher in the visual cortex, whereas the concentrations in the striatum were lower [9] (Table 5 and Fig. 7).

Four studies measured data about ataxia. NAA and its ratios were lower in all selected VOIs [5,6,7,and8]]. MSA-C, which can develop into ataxia, has shown similar differences in clinical presentation and the biomarkers, as well as their ratios [12]. A study that focused on both diseases proved changes in biomarkers over 8 years of follow-ups [5] (Table 6, Table 7).

Varying contents of tissue water (a measure of metabolite levels) may affect the metabolites in MRS [26]. As the sole metabolite that is present in the body, axons, and dendrites of neuron cells, NAA can act as the biomarker of neuronal viability and function [4]. Low NAA levels could indicate the diminution of brain tissue volume or decreased neuronal metabolism and dysfunction related to lower tCr and Glu levels. These indicate altered brain energy metabolism (tCr) and glutamatergic neuronal activity (Glu) [26] (Fig. 6). If the neuron astrocyte complex integrity is significantly disrupted, the total glutamate levels are expected to decrease, ultimately followed by atrophy [14]. Another essential biomarker is Cr peak, referred to as total creatine (tCr), which functions as an energetic marker and maintains brain energy homeostasis. The importance of tCr may refer to the limited glucose storage capability of the brain [9]. A high ratio of Cho/Cr is caused by cell membrane catabolism to compensate for the cholinergic deficit in disease progression [20]. On the other hand, ml has high levels within the glial cells, and thus, it is often taken as a marker for gliosis [4].

This review has several limitations. Diagnosing and following the development of these neurodegenerative diseases require a long examination time, which many studies lacked. Some of the retrieved studies focused on a small sample size due to the COVID-19 pandemic and the non-cooperation of elderly patients due to some degree of irritability. Additionally, different magnetic fields, VOIs, and neurochemicals impair the comparability across studies (as shown in Table 1, Table 2, Table 3, Table 4, Table 5, Table 6, Table 7). However, MRS is not considered a priority imaging technique for diagnosing these diseases yet. This can have various reasons, including conventional radiologists having problems with the analysis and interpretation of MRS signals, imprecise imaging standardization, and less-than-optimal sensitivity and specificity in clinical practice [11]. This study encourages further studies for more efficient information about biomarkers and their changes to prove the application of MRS in the prevention and early diagnosis of neurodegenerative diseases.

In line with future studies on MRS, we provide suggestions for authors.

1. Systematic reviews of nervous system disorders using MRS
2. Studying the use of MRS for cognitive pathology analysis in addiction to drugs or Internet games
3. Using the capability of MRS in studies such as the diagnosis of inflammatory diseases

## Data availability

No new data were created or analyzed during this study. Data sharing is not applicable to this article.

## Additional information

No additional information is available for this paper.

## CRediT authorship contribution statement

**Fatemeh Abbaspour:** Writing – review & editing, Project administration, Formal analysis, Data curation. **Niusha Mohammadi:** Writing – review & editing, Writing – original draft, Formal analysis, Data curation. **Hassan Amiri:** Methodology, Investigation. **Susan Cheraghi:** Writing – original draft, Project administration, Methodology, Investigation, Conceptualization. **Reza Ahadi:** Writing – review & editing, Project administration, Methodology. **Zeinab Hormozi-Moghaddam:** Writing – review & editing, Supervision, Investigation.

## Declaration of competing interest

The authors declare that they have no known competing financial interests or personal relationships that could have appeared to influence the work reported in this paper.
